# An end-to-end seizure prediction approach using long short-term memory network

**DOI:** 10.3389/fnhum.2023.1187794

**Published:** 2023-05-18

**Authors:** Xiao Wu, Zhaohui Yang, Tinglin Zhang, Limei Zhang, Lishan Qiao

**Affiliations:** ^1^School of Computer Science and Technology, Shandong Jianzhu University, Jinan, China; ^2^School of Mathematics Science, Liaocheng University, Liaocheng, China; ^3^School of Information, Yancheng Institute of Technology, Yancheng, China

**Keywords:** EEG, gamma band, epileptic seizure prediction, long short-term memory network, deep learning

## Abstract

There are increasing epilepsy patients suffering from the pain of seizure onsets, and effective prediction of seizures could improve their quality of life. To obtain high sensitivity for epileptic seizure prediction, current studies generally need complex feature extraction operations, which heavily depends on the artificial experience (or domain knowledge) and is highly subjective. To address these issues, in this paper we propose an end-to-end epileptic seizure prediction approach based on the long short-term memory network (LSTM). In the new method, only the gamma band of raw electroencephalography (EEG) signals is extracted as network input directly for seizure prediction, thus avoiding subjective and expensive feature design process. Despite its simplicity, the proposed method achieves the mean sensitivity of 91.76% and false prediction rate (FPR) of 0.29/h on Children’s Hospital Boston-MIT (CHB-MIT) scalp EEG Database, respectively, when identifying the preictal stage from the EEG signals. Furthermore, different from traditional methods that only consider the classification of preictal and interictal EEG, we introduce the postictal stage as an extra class in the proposed method. As a result, the performance of seizure prediction is further improved, obtaining a higher sensitivity of 92.17% and a low FPR of 0.27/h. The mean warning time is 44.46 min, which suggests that sufficient time is reserved for patients to take intervention measures by this prediction method.

## 1. Introduction

Epilepsy is a kind of chronic disease caused by the transient abnormal discharge of brain neurons, which leads to temporary brain dysfunction (Jordans et al., 2017). The epileptic seizures are long-term recurrent and unexpected, which could seriously damage the physical and mental health of patients. There are four main stages of an epileptic seizure, including preictal, ictal, postictal, and interictal period. Normally, patients are in the interictal state, from which if preictal phase could be identified, an imminent seizure may be avoided by medical treatment (Usman et al., 2019; Maimaiti et al., 2022).

Electroencephalography (EEG) is an important diagnostic indicator for epileptic seizures, but it is difficult to observe a significant change before a sudden onset of epilepsy from EEG signals. Therefore, the current prediction algorithms mainly focused on classification of preictal EEG and interictal EEG. It is found that EEG tends to exhibit some kinds of complex and patient-specific patterns before seizures (Herkes et al., 1993; Mormann et al., 2007). In time domain, significant differences of some statistical measures such as variance, skewness and kurtosis between preictal EEG and interictal EEG have been reported in several seizure prediction studies (Mormann et al., 2005; Aarabi et al., 2009). In the frequency domain, EEG spectral band power has been proved to be effective in classification, and has been successfully applied in prediction. In addition, connectivity measures of functional brain network based on graph theory (Rubinov and Sporns, 2010; Tsiouris et al., 2018), and some non-linear dynamical parameters such as correlation dimension (Lehnertz et al., 2001), dynamic similarity index (Quyen et al., 1999), correlation entropy and Lyapunov exponent were also extracted as features (Aarabi and He, 2017; Xu et al., 2022). However, some features have high computational complexity and lack of reproducibility and reliability (Mormann et al., 2005; Elger and Lehnertz, 2007; Assi et al., 2017; Sánchez-Hernández et al., 2022; Assali et al., 2023). Furthermore, the accuracy of these prediction algorithms needs to be improved. Usman et al. concluded that Support Vector Machines (SVM) and Convolutional Neural Networks (CNN) achieved better classification performance by comparing the results of seizure prediction approaches proposed before 2019 (Usman et al., 2019). As a popular deep learning algorithm, CNN, improved CNN and Gashis-transformer are designed for image processing (Chen et al., 2021; Zhang et al., 2021; Li et al., 2022). However, it fails to directly deal with EEG signals that are non-linear and non-stationary time series. Therefore, Truong et al. (2018) converted EEG into time-frequency graph by Short-Time Fourier Transform (STFT) as the input of CNN and obtained a sensitivity of 81.4%. CNN in this approach, however, only learns time-frequency information of EEG, which could not fully describe preictal and interictal EEG patterns. Recently, another deep learning model Long Short-Term Memory (LSTM) network has started to attract attention in seizure prediction. Because LSTM is superior in processing temporal information of brain activity, Tsiouris et al. (2018) input 643 commonly used EEG features into a LSTM network for prediction and reached 99.63% sensitivity when 1-h preictal window was used. However, deep neural networks applied in seizure prediction, regardless of CNN or LSTM, mainly play a role in classifier. If the features of preictal patterns could not be comprehensively extracted, the classification performance will be affected.

In addition, current prediction algorithms, which mainly focused on classification of preictal EEG and interictal EEG, ignored the analysis of postictal EEG. Postictal phase is a transitional period in which the brain returns to an interictal state from a seizure and it is different from the interictal phase (Büyükakr et al., 2020). Unfortunately, there is no general criterion for dividing the preictal, interictal, and postictal phase. If the postictal period is predefined for a short time, the next period of EEG may be classified as preictal state by current binary classification algorithms, which will cause false alarm in practical application. If the specified time period of postictal phase is too long, the next incoming epileptic seizure may be ignored. Consequently, the identification of a postictal state also needs to be taken into account for the prediction method. Teixeira C. et al. (2014) and Bandarabadi et al. (2015) took two classes (preictal and non-preictal period) into account for seizure prediction. Specifically, non-preictal period includes ictal, postictal, and interictal states. Furthermore, their research also considered four classes (preictal, ictal, postictal, and interictal period) to predict seizures (Teixeira C. A. et al., 2014; Direito et al., 2017). These are more reasonable than using only preictal and interictal periods.

In this paper, the filtered EEG signals are directly input into LSTM to learn deep features, which results in an end-to-end seizure prediction approach. It could not only preserve the epileptic-related information in EEG signals as much as possible, but also reduce the complexity of the manual feature extraction/design. Moreover, the identification of postictal state is taken into account in this study. Despite its simplicity, the proposed method tends to achieve encouraging performance in seizure prediction.

The remainder of the work is organized as follows. In section “2. Materials and methods,” we introduce the information of database used in this paper and the proposed scheme, respectively. In addition, performance evaluation and seizure prediction are shown in this section. In section “3. Results,” we present experiments results. In section “4. Discussion,” we discuss different seizure prediction methods in the same data set. Finally, we conclude this paper in section “5. Conclusion.”

## 2. Materials and methods

### 2.1. EEG dataset

The Children’s Hospital Boston-MIT (CHB-MIT) scalp EEG Database (Shoeb, 2010) is the only open dataset with long-term continuous scalp EEG recordings which have been applied by many studies for evaluation of seizure prediction algorithms (Zandi et al., 2010; Myers et al., 2016; Khan et al., 2017; Detti et al., 2018; Usman et al., 2021). The EEG data were collected from 22 pediatric subjects including 5 males and 17 females. All EEG signals were sampled at the rate of 256 Hz and most of them were recorded by 23 electrodes with bipolar montage according to International 10–20 System. The start and end time of each seizure were manually annotated by clinical experts with visual inspection. More detailed description could be found on www.physionet.org.

Some seizures which are very close in time were excluded, because this study only focuses on prediction of leading seizures that occur more than 60 min after the previous seizure. There is no gold standard for defining the length of four stages (preictal, ictal, postictal, and interictal stage). In order to avoid any potential contamination between interictal and preictal EEG signals, interictal EEG recordings are selected from at least 5 h after an epileptic seizure and 3 h before the next seizure. The preictal period covered 1 h prior to each leading seizure. Ictal state is annotated by clinical experts. The period within 1 h after the ictal phase is defined as a postictal phase. Based on these considerations and definitions, 13 patients have sufficient data for analysis of preictal and interictal state, which are shown in Table 1. Sufficient postictal EEG could only be obtained from 7 out of the 13 subjects.

**TABLE 1:** Details of the CHB-MIT dataset used in this work.

Subjects	Gender	Age (Years)	No. of channels	No. of seizures	Duration of recordings (hh: mm: ss)
1	F	11	23	6	40:33:08
2	M	11	23	3	35:15:59
3	F	14	23	4	38:00:06
5	F	7	23	5	39:00:10
7	F	14.5	23	3	67:03:08
9	F	10	23	3	67:52:18
10	M	3	23	6	50:01:24
14	F	9	23	3	26:00:00
18	F	19	23	4	29:55:46
20	F	6	23	4	27:36:06
21	F	13	23	4	32:49:49
22	F	9	23	3	31:00:11
23	F	6	23	3	26:33:30
Total	–	–	–	51	511:41:35

Gender: female (F), male (M). No. of channels: number of EEG channels included. No. of seizures: number of leading seizures.

### 2.2. Methods

#### 2.2.1. Pre-processing

All the raw EEG signals are split into samples by a 5-s time window without any overlap. In order to test the minimum time prediction window (MTPW), EEG data is also segmented by a variable window with the length varies from 5 to 150 s (i.e., the number of samples included in each segment is the window length divided by five.). Samples containing significant EMG interference were directly removed. Five standard EEG frequency bands [i.e., δ (0-4 Hz), θ (4-8 Hz), α (8-13 Hz), β (13-30 Hz), and γ (30-128 Hz)] were extracted by finite impulse response (FIR) band-pass filter.

#### 2.2.2. Classification

**FIGURE 1:**
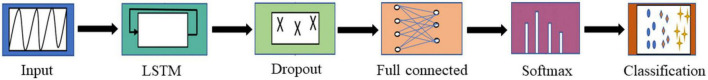
The architecture of LSTM network.

The filtered EEG data of each frequency band is directly input into LSTM for seizure prediction. First, we only consider the preictal and interictal EEG as previous studies that also use the CHB-MIT dataset, so that we can compare their prediction performance easily. Then, the postictal period, as the third class, is included in the classification task to predict seizures. Both the binary and multiple classification tasks use the same method framework, as shown in Figure 1.

As aforementioned, we utilize LSTM model to classify considering its advantage in memory function. Concretely, as a modified Recurrent Neural Network (RNN), LSTM solves the problems of gradient explosion, gradient vanishing and the long-term dependence to some extent by introducing gate mechanism (input gate, forget gate and output gate) and cell state (Malhotra et al., 2015; Hefron et al., 2017). The forget gate determines how much of the information stored in the previous cell state needs to be deleted. The input gate decides what new information is stored in current cell state. The output gate determines what information is to be output from the cell state. The transmission of information across the cell state that contains long-term and short-term memory allows the LSTM to learn about the temporal dependence of the sequence.

The LSTM network architecture is shown in Figure 1. It is constructed with input layer, LSTM layer, dropout layer, fully connected layer, SoftMax layer and classification layer. The size of the input layer is 1280 × 23 (i.e., the length of EEG sequence × the number of channels), and the LSTM layer is composed of 128 memory units. The dropout layer inserted after LSTM with the probability of 0.5 is used to prevent overfitting and accelerate the training speed. The output size of a fully connected layer depends on the number of classes.

The data of each subject was divided into two parts. The first part, containing the first two seizures was used for the training set in order to optimize the parameters of the classifiers. The second part including the remaining seizures was used as the testing set. It is noted that the training aims to simulate a real-world situation where a predictor has to be developed based on the first two seizures in clinic environment. However, epileptic seizures can cause certain damage to the brain, which leads to the basic state of the pre-epileptic interictal phase and the post-epileptic interictal phase are different. Therefore, using the preictal and interictal EEG signals of the first two seizures may not predict the subsequent seizures. The EEG signals of the preictal and postictal state have certain similarity. Therefore, postictal state can be used as a new baseline for subsequent seizures. That is, the interictal and postictal state of the first two seizures can be used to predict subsequent seizures.

Due to the amount of interictal EEG is much larger than that of preictal EEG and postictal EEG, we randomly select samples from the interictal class with the same number as preictal or postictal samples, which mitigates the class imbalance problem. Since the available data is limited for deep learning, a 10-fold cross validation strategy was adopted during training to prevent the LSTM model from overfitting. Ten percent of the training data is randomly selected as a validation set to monitor the training process.

#### 2.2.3. Performance evaluation and seizure prediction

In order to demonstrate the performance of this prediction method more comprehensively, results are provided by two ways described as follows.

##### 2.2.3.1. Segment-based evaluation

All EEG samples for each subject are put together to test the performance of this prediction algorithm by using a 10-fold cross validation. Specifically, under optimal performance conditions, a segment would be predicted as preictal EEG if over half of the number of samples in this segment were classified as preictal in the classification of preictal and interictal EEG. Similarly, if more than 1/3 of the samples in a segment are identified as preictal when using the classification of preictal, postictal and interictal EEG, it is considered to be from the preictal phase.

For the classification of preictal and interictal EEG, the measures for evaluating seizure prediction performance are sensitivity and specificity which are defined by equation (1) and (2). The true positive (TP) is the number of correctly classified preictal segments, and true negative (TN) is the number of true interictal segments that are identified by classifier. Similarly, FP is false positive which is the number of EEG segments that are incorrectly identified as preictal segments and false negative (FN) is the number of preictal class predicted as interictal class. For multi-classification, the true positive rate (TPR) of each class is used as evaluation index.


(1) $$S\,e\,n\,s\,i\,t\,i\,v\,i\,t\,y=\frac{T\,P}{T\,P+F\,N}$$

(2) $$S\,p\,e\,c\,i\,f\,i\,c\,i\,t\,y=\frac{T\,N}{T\,N+F\,P}$$

##### 2.2.3.2. Event-based evaluation and seizure prediction

**FIGURE 2:**
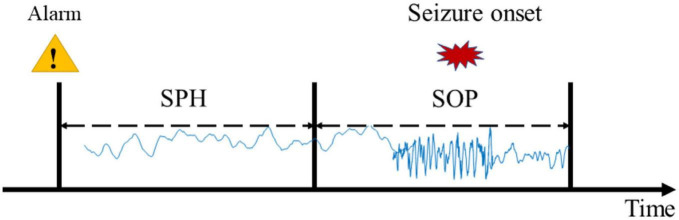
Definition of the seizure occurrence period (SOP) and the seizure prediction horizon (SPH).

Considering the limited seizures for each subject, we used leave-one-out cross-validation. Concretely, if the subject had *N* seizures, the (*N* − 1)seizures would be used for training and the remaining one would be used for testing. This round is done *N* times, and the final result of this subject is the average of *N* times. To improve the reliability of the prediction, a prediction window of 10 min was applied. According to experiential knowledge, if more than 70% of EEG samples during 10 min continuous recordings are identified as preictal, the warning alarm would be raised (Truong et al., 2018; Wei et al., 2019). The indexes used in this study are seizure occurrence period (SOP) and seizure prediction horizon (SPH) proposed by Maiwald et al. (2004) (see Figure 2). SPH is a predefined interval between the first alarm and the incoming seizure, which is also a period reserved for patients to take intervention measures. SOP is the period during which a seizure is expected to occur. Therefore, for a correct prediction, seizure would not occur during the SPH and must occur within the SOP. Otherwise, it’s a false alarm. There are no common criteria for the length of SOP and SPH, but the SPH should be long enough for intervention and the SOP should not be too long in case of patient’s anxiety. We also consider the sensitivity and specificity of the prediction algorithm at different time intervals to optimize the prediction accuracy and reduce false positives. So, we define the seizure prediction horizon (SPH) of 30 min and the seizure occurrence period (SOP) of 20 min to achieve better predictive performance.

For event-based evaluation, sensitivity is defined as the number of correctly predicted seizures divided by the total number of seizures. False prediction rate (FPR) is quantified as the number of false alarms per hour. Warning time refers to the period of time before a seizure occurs when physiological changes in the nervous system reach a level that indicates a seizure may occur. In other words, the warning time is the interval between the first alarm and the incoming seizure onset.

To evaluate the statistical significance of the predictor, we also compare our seizure prediction performance with that of a random predictor. Given an FPR, the probability to raise an alarm in an SOP can be approximated by Schelter et al. (2006):


(3) $$P\approx 1-e^{-F\,P\,R\cdot S\,O\,P}$$

Therefore, the probability for predicting at least *m* of *M* independent seizures by chance is given as follows:


(4) $$p=\sum_{i\geq m}\left(\begin{array}{c} M\\ i \end{array}\right)\,P^{i}\,{\left(1-P\right)}^{M-i}$$

We calculate *p* for each subject using the FPR of that subject and the number of seizures (*m*) predicted by our method. If *p* is less than 0.05, we can conclude that our prediction method is significantly better than a random predictor at a significance level of 0.05.

## 3. Results

### 3.1. Classification of preictal and interictal EEG

#### 3.1.1. Segment-based results

**FIGURE 3:**
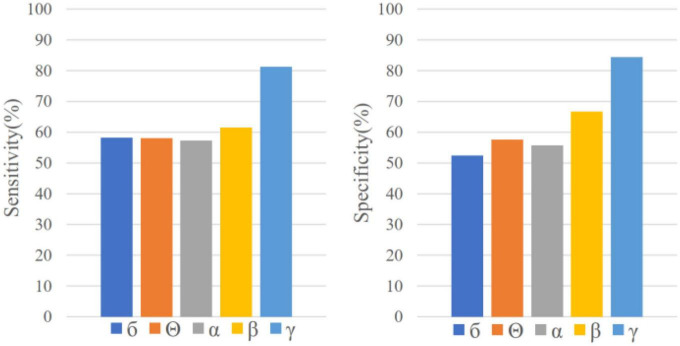
Sensitivity and specificity in five frequency bands for sample-based evaluation.

EEG samples of five frequency bands *(δ, θ, α, β *and* γ)* are input into LSTM, respectively, for seizure prediction. As is shown in Figure 3, the sensitivity and specificity of γ band is the highest, which indicates that it is more distinguishable between preictal state and interictal state in γ band with this prediction method. Therefore, EEG signals in band γ were selected for prediction in this study.

**FIGURE 4:**
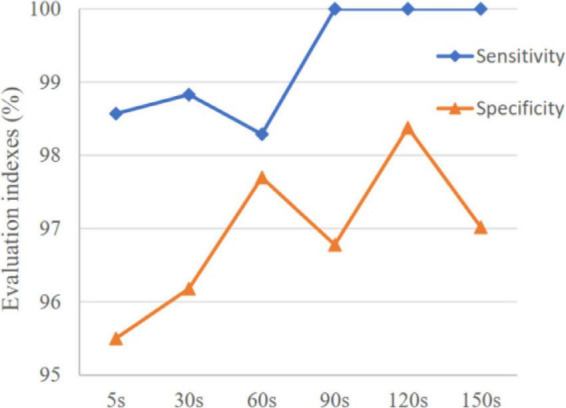
Variation of mean sensitivity and specificity with increasing prediction window.

The variation of mean sensitivity and mean specificity with increasing prediction window are shown in Figure 4 and the detailed results of each subject can be found in Table 2. It is illustrated that sensitivity and specificity are gradually improved with the increase of time window and reach a plateau after 120 s. Consequently, the MTPW of this prediction approach was 120 s under which the mean sensitivity could reach 100% and mean specificity was 98.38%.

**TABLE 2:** Seizure prediction results based on segments under different time prediction windows.

Subjects	No. of seizures	5 s		30 s		60 s		90 s		120 s		150 s	
		SEN (%)	SPE (%)	SEN (%)	SPE (%)	SEN (%)	SPE (%)	SEN (%)	SPE (%)	SEN (%)	SPE (%)	SEN (%)	SPE (%)
1	5	99.99	99.86	100	100	100	100	100	100	100	100	100	100
2	3	99.86	99.43	100	100	100	100	100	100	100	100	100	100
3	4	99.44	99.11	100	100	100	100	100	100	100	100	100	100
5	4	100	90.13	100	90.48	100	100	100	95.24	100	100	100	88.73
7	3	97.18	99.27	97.22	100	100	100	100	97.06	100	100	100	100
9	5	94.98	96.33	95.24	98.15	96.88	100	100	100	100	100	100	100
10	7	97.74	92.34	97.22	94.44	100	94.74	100	100	100	94.74	100	94.44
14	3	86.61	82.20	91.98	81.82	100	100	100	100	100	100	100	100
18	3	99.89	99.77	100	100	100	100	100	100	100	100	100	100
20	5	94.18	96.81	95.12	94.34	80.95	100	100	92.86	100	100	100	100
21	4	98.55	78.55	100	83.33	100	85.33	100	78.57	100	92.96	100	83.33
22	3	99.64	92.51	100	90.67	100	90	100	94.44	100	91.3	100	94.74
23	3	100	99.43	100	100	100	100	100	100	100	100	100	100
**Average**		**97.55**	**94.29**	**98.21**	**94.86**	**98.29**	**97.70**	**100**	**96.78**	**100**	**98.38**	**100**	**97.02**

SEN, sensitivity; SPE, specificity.

#### 3.1.2. Event-based results

For event-based evaluation, the proposed method achieved the sensitivity of 91.76% and FPR of 0.29/h on average, as presented by Table 3. Except for Subjects 10, 14, 20, the *p*-value indicates that our prediction method is significantly superior to a random predictor. For each patient, most seizures were successfully predicted and the warning time was more than 48 min. It indicated that there was enough time for patients to take intervention measures for the incoming seizure, because of the high sensitivity and timeliness of this approach.

**TABLE 3:** Event-based evaluation results.

Subjects	No. of seizures	Sensitivity (%)	Warning time (Min)	FPR (/h)	Testing duration (h)	*p*
1	6	96.12	46.60	0.16	18	0.035
2	3	78.00	45.75	0.32	25	0.029
3	4	100	49.86	0.21	24	0.025
5	5	81.78	47.19	0.20	17	0.037
7	3	78	48.07	0.24	8	0.016
9	3	100	49.92	0.25	6	0.018
10	6	98.50	45.07	0.24	21	0.072
14	3	100	48.75	0.50	2	0.063
18	4	83.15	45.70	0.28	21	0.042
20	4	98.13	49.84	0.30	13	0.047
21	4	88.36	49.89	0.40	5	0.078
22	3	90.83	48.35	0.29	14	0.008
23	3	100	49.92	0.38	13	0.040
**Average**		**91.76**	**48.07**	**0.29**		

FPR, false prediction rate; *p*, *p*-value.

### 3.2. Classification of preictal, interictal, and postictal EEG

**FIGURE 5:**
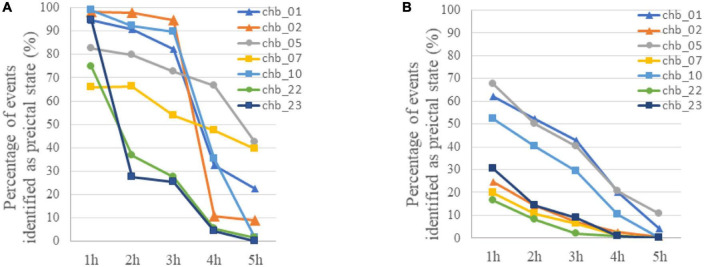
**(A)** The variation of classification results with time after the ictal phase when LSTM was trained using only preictal and interictal EEG. The percentage of samples identified as preictal EEG for each subject was calculated with event-based classification. **(B)** The variation of classification results with time after the ictal phase when LSTM was trained using preictal, interictal and postictal EEG. The percentage of samples identified as preictal EEG for each subject was calculated with event-based classification.

In addition to preictal and interictal state, prediction method based on binary classification may be used in the postictal state when a seizure is failed to be predicted in practice, and the variation of event-based classification results with time after the ictal phase was tested. As is shown in Figure 5A, most of EEG samples in the first hour after ictal state (postictal state) are identified as preictal for 7 subjects, and then an increasing number of samples were classified as interictal with time. In four of the subjects (chb_01, chb_02, chb_05, and chb_10), more than 70% of the EEG samples on average were still identified as preictal in the third hour after the ictal phase. Accordingly, there would be many false alarms for a period after seizure onset.

For these reasons, a prediction method based on the classification of preictal, interictal and postictal EEG was proposed. The TPR of each class in segment-based evaluation with MTPW of 120 s and results of event-based evaluation are shown in Table 4. Except for Subject 1, it shows that our prediction method is significantly superior to a random predictor. The average TPR of preictal, interictal and postictal EEG were 98.64, 98.39, and 92.65%, respectively, which indicated that all three brain states could be identified by the proposed approach. The sensitivity for event-based evaluation was 92.17%, namely, almost all seizures could be correctly predicted with this method as well. Compared with results of the classification of preictal and interictal EEG, the number of EEG samples incorrectly predicted as preictal EEG decreases for all subjects after seizure onset, as displayed in Figure 5B. Meanwhile, FPR was 0.27/h (i.e., the average of chb_01, chb_02, chb_05, chb_07, chb_10, chb_22, and chb_23), and the mean warning time could achieve 44.46 min as well.

**TABLE 4:** Seizure prediction results based on multi-classification.

Subjects	No. of seizures	Segment-based			Event-based				
		Preictal (%)	Interictal (%)	Postictal (%)	Sensitivity (%)	FPR (/h)	Testing duration (h)	Warning time (min)	*p*
1	5	100	100	91.67	100	0.47	17	41.67	0.156
2	3	100	100	90.00	85.50	0.30	24	47.09	0.025
5	4	100	95.92	100	92.00	0.18	16	43.41	0.018
7	3	94.64	97.19	95.99	81.05	0.26	7	41.08	0.019
10	5	94.44	100	83.33	95.50	0.23	20	44.25	0.046
22	3	100	97.83	87.5	96.00	0.30	13	45.98	0.026
23	3	100	100	92.68	95.12	0.16	12	47.17	0.008
**Average**		**98.44**	**98.71**	**91.60**	**92.17**	**0.27**	**–**	**44.46**	

## 4. Discussion

As an important part of previous prediction algorithms, feature engineering is closely related to the accuracy of prediction. However, it is a challenging process of finding discriminative features for a preictal state from other seizure states. In our seizure prediction method, features are learned automatically by LSTM network instead of being searched manually. The two important network parameters, the number of memory units and dropout rate, have already been optimized by Tsiouris et al. (2018). It is verified that the classification accuracy improved with increasing number of memory units. Considering the limitations of computer hardware, the maximum number of memory units is chosen as 128. We have proved that dropout rate only has marginal effects on classification results within an appropriate range (<0.8) which is consisted with the results of Tsiouris et al. (2018). Although epileptic seizures have specific effects on different patients, the proposed method with optimized parameters could work well on all subjects, which indicates that the commonality of preictal EEG among all subjects is extracted and there is no need for specific parameter optimization in practical application. Significantly, postictal state is taken into account in this study, because the algorithm would be applied to postictal EEG if a seizure is failed to be predicted. Furthermore, the postictal period is specific for each patient, which could not be simply ignored by the prediction algorithm. The method presented in this study is easy to implement and has good reproducibility. It works well for the Children’s Hospital Boston-MIT EEG dataset.

For segment-based evaluation, the sensitivity of this prediction algorithm based on binary classification is the highest compared to previous studies, as shown in Table 2. Furthermore, the mean sensitivity and specificity could reach 97.55% and 94.29% even in 5 s prediction window and are improved to 100 and 98.38% with 120 s time window (see Table 2), which illustrates the good responsiveness of this method. For event-based evaluation, the sensitivity can reach 91.76% and the FPR is 0.29/h. When the testing set consists of interictal EEG and preictal EEG like other studies (Alotaiby et al., 2017; Truong et al., 2018; Tsiouris et al., 2018; Tang et al., 2020), the FPR can be reduced from 0.29/h to 0.27/h. As shown in Table 6, the performance of our method is better than that of most other studies. Concretely, the sensitivity of our proposed method is 10% higher than the approach presented by Truong et al. (2018). In addition, Tsiouris et al. (2018) achieved the best sensitivities for both segment-based and event-based evaluations. However, the parameters of LSTM were optimized for each subject, and authors also pointed out that the FPR could reach 0.14/h without LSTM architecture tuning individually. Additionally, it is controversial that the authors claimed EEG data from all subjects were applied, whereas 60 min of continuous preictal EEG before some seizures (non-leading seizures) could not be obtained from raw data. Similarly, Zhang and Parhi (2016) performed specific feature selection for each patient as well and had a low FPR of 0.05/h. These, however, require sufficient expertise and time to complete feature engineering when the predication methods are used for a new patient.

For clinical consideration, SPH should be long enough for patients to take measures to prevent the incoming seizures, while SOP should be as short as possible to relieve patients’ anxiety during waiting (Maiwald et al., 2004). In general, the warning time (i.e., the interval between the seizure and the warning alarm) must be long enough for intervention but not too long in case of anxiety. Some studies implicitly used the SPH of 0 min (i.e., if an alarm occurs during a preictal period, it is considered as a successful prediction), which could have overestimated the prediction performance. As shown in Table 3, the mean warning time of our method could reach 48 min, which means there is sufficient time for patients to take intervention measures. Furthermore, the time of alarm could be flexibly set according to the needs of patients in practical applications. For example, patients who are well cared for, could set a long prediction window to reduce anxiety and improve the reliability of prediction. Whereas, elderly patients who live alone with limited mobility should set a short prediction window to ensure adequate intervention time.

The prediction performance, to a certain extent, is improved by the classification of preictal, interictal, and postictal EEG prediction algorithm. In addition to all seizures being successfully predicted, FPR is also reduced. Because postictal EEG is involved in training, the number of postictal samples incorrectly predicted as preictal EEG decreases for all subjects, compared to the classification of preictal and interictal EEG algorithm (see Figure 5B). But the FPR of most subjects are higher in postictal period than interictal period. It suggests that there are certain similarities in gamma band between EEG modes before and after the same seizure. As illustrated in Figures 5A, B, the percentage of samples falsely identified as preictal EEG is high for subject chb_01, chb_05, and chb_10 in the first 3 h after seizures by using both prediction methods, hence it cannot exclude the possibility that the three patients may have a long recovery time after a seizure.

In some cases, such as a short interval between seizures, failing to predict or suppress the incoming seizure, postictal EEG would be included in the classification. Therefore, identification of postictal state needs to be taken into account. Meanwhile, as mentioned earlier, different subjects and even different seizures have specificity, it is necessary to carry out relevant research on adaptive division of different stages to predict seizures successfully. According to the comparison results of Tables 5, 6, LSTM deep learning is an effective technique for seizure prediction. CNN is typically used for image data, and SVM is typically used for classification tasks. LSTM is designed to overcome the vanishing gradient problem, which is a common issue in recurrent neural networks that can make it difficult to learn long-term dependencies in sequential data. In contrast, CNN and SVM are not designed specifically for sequential data, and may struggle to capture long-term dependencies. Since EEG data is long-term dependent data, LSTM-based prediction method is better than other methods. The prediction performance may be improved, if an adaptive extraction method for the interested frequency band could be introduced before input into LSTM, which could be another research direction in the future. In addition, due to the small number of subjects and seizures, only predictor development can be implemented on the CHB-MIT database. In order to meet the practical application requirements, we should refer to the proposal of Teixeira C. A. et al. (2014) to train the LSTM network using the data from the previous limited number of seizures, and then make predictions for the subsequent seizures. Therefore, it is necessary to verify the reliability of our proposed algorithm on a long-term database in the future work.

**TABLE 5:** Comparison of seizures prediction methods using CHB-MIT dataset for segment-based evaluation.

References	No. of subjects	No. of seizures	Features	Classifier	SEN (%)	SPE (%)	Preictal duration (min)	MTPW (s)
Cho et al., 2016	21	65	Phase locking value	SVM	82.44	82.76	5	1
Tsiouris et al., 2018	24	185	Statistical moments, zero crossings, Wavelet Transform coefficients, cross-correlation, PSD, graph theory	LSTM	99.63	99.78	60	50–250
Zhang et al., 2020	19	–	Pearson correlation coefficient	CNN	92.90	87.04	15	8
**Our work**	**13**	**51**	**–**	**LSTM**	**100**	**98.38**	**60**	**120**

**TABLE 6:** Comparison of seizures prediction methods using CHB-MIT dataset for event-based evaluation.

References	No. of subjects	No. of seizures	Features	Classifier	SEN (%)	FPR (/h)	Preictal duration (min)	Warning time (min)
Zandi et al., 2010	3	18	Zero crossings, similarity dissimilarity index	–	83.81	0.17	40	22.5
Myers et al., 2016	10	30	Phase/amplitude locking value	–	77.00	0.17	40	2–62
Chu et al., 2017	13	125	Fourier transform coefficients, PSD	–	83.33	0.39	86	45.3
Truong et al., 2018	13	64	STFT spectral images	CNN	81.20	0.16	5	–
Khan et al., 2017	15	18	Wavelet transform coefficients	CNN	83.33^a^	0.15	10	6
Alotaiby et al., 2017	24	170	Common spatial pattern statistics	LDA	81.00	0.47	60	38.35
Büyükakr et al., 2020	10	62	Statistical, spectral moments	MLP	89.81	0.08	30	–
**Our work**	**13**	**51**	**–**	**LSTM**	**91.76**	**0.29**	**60**	**48.07**

^a^The study implicitly used a zero SPH without any clinical consideration and the performance may be overestimated.

The authors claimed EEG data from all subjects were applied, whereas 60 min of continuous preictal EEG before some seizures could not be obtained from raw data.

## 5. Conclusion

Deep learning algorithms, in increasing studies, have demonstrated their abilities to deal with non-stationary and chaotic EEG signals, which open new opportunities for challenging medical applications like epileptic seizure prediction. In this study, an end-to-end seizure prediction approach based on LSTM is proposed to classify preictal, postictal and interictal EEG. Although it is simple, the prediction method provided high sensitivity and low FPR without complex feature engineering techniques. Additionally, by introducing the postictal stage into the classification task, the performance is further improved. In the future, we plan to test the proposed approach on a large amount of clinical data, and the adaptive division of different seizure stages needs to be further investigated.

## Data availability statement

The original contributions presented in this study are included in the article/supplementary material, further inquiries can be directed to the corresponding authors.

## Author contributions

ZY: software. LZ: supervision. XW: writing—original draft. TZ and LQ: writing—review and editing. All authors contributed to the article and approved the submitted version.
